# The deposition and characterization of starch in *Brachypodium distachyon*

**DOI:** 10.1093/jxb/eru276

**Published:** 2014-07-23

**Authors:** Vanja Tanackovic, Jan T. Svensson, Susanne L. Jensen, Alain Buléon, Andreas Blennow

**Affiliations:** ^1^Department of Plant and Environmental Sciences, University of Copenhagen, Copenhagen, DK-1871, Denmark; ^2^Nordic Genetic Resource Centre, P.O. Box 41, SE-230 53 Alnarp, Sweden; ^3^UR1268 Biopolymeres Interactions Assemblages, INRA, F-44300 Nantes, France

**Keywords:** Barley, *Brachypodium distachyon*, endosperm, grass domestication, starch biosynthesis, starch granule.

## Abstract

*Brachypodium distachyon* is a non-domesticated cereal. Nonetheless, *Brachypodium* was recently introduced as a model plant for temperate cereals. This study compares grain starch metabolism in *Brachypodium* and barley (*Hordeum vulgare*). In *Brachypodium*, we identified and annotated 28 genes involved in starch metabolism and identified important motifs including transit peptides and putative carbohydrate-binding modules (CBMs) of the families CBM20, CBM45, CBM48, and CBM53. Starch content was markedly lower in *Brachypodium* grains (12%) compared to barley grains (47%). *Brachypodium* starch granules were doughnut shaped and bimodally distributed into distinct small B-type (2.5–10 µm) and very small C-type (0.5–2.5 µm) granules. Large A-type granules, typical of cereals, were absent. Starch-bound phosphate, important for starch degradation, was 2-fold lower in *Brachypodium* compared with barley indicating different requirements for starch mobilization. The amylopectin branch profiles were similar and the amylose content was only slightly higher compared with barley cv. Golden Promise. The crystallinity of *Brachypodium* starch granules was low (10%) compared to barley (20%) as determined by wide-angle X-ray scattering (WAXS) and molecular disorder was confirmed by differential scanning calorimetry (DSC). The expression profiles in grain for most genes were distinctly different for *Brachypodium* compared to barley, typically showing earlier decline during the course of development, which can explain the low starch content and differences in starch molecular structure and granule characteristics. High transitory starch levels were observed in leaves of *Brachypodium* (2.8% after 14h of light) compared to barley (1.9% after 14h of light). The data suggest important pre-domesticated features of cereals.

## Highlight text

A thorough study of starch biosynthesis and deposition in a non-domesticated wild grass was performed using *Brachypodium distachyon* as a model.

## Introduction

Starch is the most important source of carbohydrates for humans, and starch from cereals is of crucial value. Worldwide, starch produced by cereal crops has provided the most important human dietary energy for millennia. In 2012, world production of cereals amounted to 2.3 billion tons (Food and Agriculture Organization, FAO, 2012: http://www.fao.org/docrep/018/al999e/al999e.pdf).

Starch is the principal storage product of the majority of plants. It is stored in both photosynthetic and non-photosynthetic organs, among which the grass grain is of central importance, with starch forming its main constituent. Starch is synthesized in well-organised starch granules in amyloplasts in the grain tissue and is made up of two polysaccharides: amylose and amylopectin. Amylose makes up 25–30% of the starch granule, possesses an α-1,4 linkage backbone structure and can be sparsely branched via α-1,6 linkages. Amylose is probably mostly amorphous in the starch granule. Amylopectin typically comprises 70–75% of the starch granule; it is more than 100-fold larger than amylose and contains clustered α-1,6 linkages. Clustering of the amylopectin chains in the granule results in 9-nm alternating branched amorphous and more-linear crystalline, double-helical lamellae (Damager *et al*., 2010; Pérez and Bertoft, 2010). Based on X-ray powder diffraction, all normal cereal starch granules show the so-called A-type crystalline polymorph, which distinguishes cereal storage starch from tuberous storage starch, which is often of the B-type crystalline polymorph. Starch is also slightly phosphorylated (0.1–1% phosphate w/w) which acts as a signal for starch remobilization (Blennow and Engelsen, 2010), but interestingly cereal endosperm starch has very low starch-phosphate content (0.01–0.06% phosphate w/w) as compared to tuberous storage starch and transitory leaf starch (Lim *et al*., 1994; Blennow *et al*., 2000b, 2002). At the micrometre level, starch assembles into large, distinctly shaped granules of which the form and topography vary tremendously between different plants and organs (Jane *et al*., 1994).

For dietary needs, large grains are of major importance because grain size is linked to large amounts of its main component, starch, and hence large grains provide superior nutritional quality (Blennow *et al.*, 2013). Nutritional quality has been, and still is, of main importance for human and animal consumption. In the human diet, the dominating importance of grain starch is reflected in the tremendous efforts over millennia to improve starch yield and quality by selection, breeding, and very recently by directed *in planta* biotechnological modification (Blennow *et al.*, 2013). However, additional quality traits are now demanded that are not linked to high dietary energy, but instead to more complex health-associated traits, such as high dietary fibre and antioxidant content. Many such traits can probably be found in wild progenitors of modern cereals and cereal landraces, making them useful as value-added biopolymers. Knowledge about starch deposition and structure in wild grasses is not extensive, and the genetic capacity for biosynthesis of these new high-value grain components and the composition of ancient and wild grains are insufficiently characterized (Shapter *et al.*, 2008).

It is well known that ancient collected grasses were low yielding and performed suboptimally. The direct human selection of grasses with superior grain led to domestication, but as the initial population that contributed to domesticated plants was small, ‘domestication bottleneck’ effects were unavoidable. This implies loss of genetic diversity and has inevitably resulted in a reduction of genetic factors for potentially important wild traits in domesticated crops compared to their wild ancestors (Doebley, 1989). Likewise, retained alleles have been fixed for desirable traits and these phenotypes became typical across a broad range of domesticated plants. A common suite of these fixed traits is a marker of domestication, known as the ‘domestication syndrome’ (Hammer, 1984; Doebley *et al.*, 2006; Meyer *et al.*, 2012). The suite includes a wealth of traits among which large grains, a non-brittle rachis, local abundance, annuality, shorter seed dormancy, polyploidy, harvestability, and relative ease of seed dehulling have been prioritized (Harlan *et al.*, 1972). The full consequences of the extent of genetic bottlenecks and reduced diversity among domesticated and wild grasses cannot be evaluated based on present knowledge, but wild relatives present an interesting reference system for such investigations. Potentially valuable lost traits can be transferred back into domesticated cereals from wild progenitors.

*Brachypodium distachyon* (L.) P. Beauv. is a wild grass that has recently been established as a model system for temperate grasses. *Brachypodium* remnants have been recovered from a ground stone artefact from the Upper Palaeolithic site of Ohalo II in Israel (Piperno *et al.*, 2004), which reveals that this grass was used in human diet while humans were gathering grains for food. However, there is no evidence that *Brachypodium* has ever been cultivated and finally domesticated. Several traits of *Brachypodium* such as suboptimal harvestability due to shattering, needle-shaped grains, small plant and grain size, and low-glycemic grain composition support this conclusion that ancestral humans did not consider *Brachypodium* as a target for domestication. Considering that *Brachypodium* was the first wild grass with a sequenced genome (Vogel *et al.*, 2010) and developed genetic tool box (Mur *et al.*, 2011), as well as being able to be propagated quickly, it is a valuable model for faster biotechnological screening of novel and undomesticated cereal traits (Bevan *et al.*, 2010). Recent analyses of the *Brachypodium* grain have revealed features including high protein and (1–3)(1–4)-β-glucan (BG) content (Larré *et al.*, 2010; Guillon *et al.*, 2011; Opanowicz *et al.*, 2011; Hands and Drea, 2012). Such *Brachypodium* features, together with access to the sequenced genome (Vogel *et al.*, 2010), and developed genetic tools make it a valuable model for further improving modern cereals (Draper *et al.*, 2001; Garvin, 2007; Garvin *et al.*, 2008; Brkljacic *et al.*, 2011; Vain, 2011).

Here we present the first investigation on diversity of starch in grasses. We focussed on the biosynthetic capacity, structure, and composition of starch in the *Brachypodium* grain. We found distinct differences as well as conserved features in starch biosynthesis capacity, gene expression, and starch structure between *Brachypodium* and its domesticated relative barley (*Hordeum vulgare* L. ssp. *vulgare*). They indicate both important conserved molecular features of starch and its metabolism as well as innovations related to domestication of cereal grain from wild grasses. Specifically, *Brachypodium* grain displayed low starch content, small granular and amorphous starch granules, and high cell wall and protein content. Distinct features could be linked to specific differences in starch biosynthesis enzyme expression profiles.

## Materials and methods

### Identification of genes and domain structure

Sequences were collected from the National Centre for Biotechnology Information database (NCBI, http://www.ncbi.nlm.nih.gov/). Sequence similarity searches were performed by BLASTP with standard settings in the *Brachypodium* genome database (http://www.brachypodium.org/) and in Phytozome (http://www.phytozome.org). Sequences with high percentage identity were selected to represent putative *Brachypodium* starch enzymes. Correct members of each of the gene classes were confirmed by BLASTP using the NCBI non-redundant database. Additional alignments were done using ClustalW Omega (http://www.ebi.ac.uk/Tools/msa/clustalo/). Alignment and phylogeny relationships were done with MEGA 5 (Tamura *et al.*, 2011). A phylogenetic tree was constructed using the neighbour-joining method. Putative domains were identified using the Pfam database (http://pfam.sanger.ac.uk/). The tandem domain CBM45 was identified manually, using available sequences (Glaring *et al.*, 2011). Signal sequences were identified in Signal 3 (http://www.cbs.dtu.dk/services/SignalP/).

### Gene expression

Transcriptome data for starch biosynthetic genes for *Brachypodium* and barley were extracted from previously published data sets obtained from two independent studies: Davidson (2012) and Radchuk (2010), respectively. A *Brachypodium* gene expression matrix was generated using RNA-Seq Illumina technology, while barley mRNA expression profiles were collected in a cDNA macroarray dataset. Data from barley were unlogged and transformed to relative values (fold change) to allow identification of expression values, and expression trends for starch-related genes in both species were analysed individually.

### Plant material

*Hordeum vulgare* ssp. *vulgare* cv. Golden Promise (spring barley) and *Brachypodium distachyon* (L.) P. Beauv. (purple false brome) lines 21 (Bd21) and 21–3 (Bd21-3) were used throughout this study. Bd21 had the first sequenced genome of *Brachypodium* and Bd21-3 is superior for genetic transformation. Plants were grown in a temperature-controlled greenhouse (22°C day/16°C night) at ambient light levels. Seed samples of *B. distachyon* were a gift from Dr John Vogel, USDA-ARS, USA.

### Starch extraction

Grains were harvested from plants and ground to a fine powder with a tissue lyser (TissueLyser, Retsch, Qiagen). Starch was isolated as described in Carciofi *et al.* (2011), with additional sieving through a 36 µm mesh. The preparation was inspected by microscopy to evaluate purity and damaged granules and only preparations with less than 1% damaged granules were kept for further analysis.

Extraction of starch granules from leaves was performed as described by Zeeman *et al.* (1998) with minor modifications. Five-week-old *Brachypodium* and barley leaves (3g) were collected after a 10h light period, immediately frozen in liquid nitrogen and stored at –80°C. Frozen leaves were homogenized using a Polytron PT 3000 Blade-type Homogenizer in 35ml of 100mM 3-(N-morpholino)propane sulfonic acid (MOPS), pH 7.2, 5mM EDTA, and 10% (v/v) glycerol. The homogenate was filtered through two layers of cloth and centrifuged for 10min, at 4°C (3000 *g*). The pellet was suspended and washed twice in 30ml of the same medium including 0.5% (w/v) SDS. A layer on the top of a dense debris pellet was identified as starch granules, which were collected with a pipette, and washed twice with SDS-containing medium and six times with ddH_2_O to completely remove debris. Sedimented starch was inspected visually for purity and granule quality, and then air dried at room temperature.

### Total starch analysis

Mature grains were powdered with a tissue lyser, and the powder was dried by lyophilization overnight; 10mg samples were used for analysis. Soluble sugars were removed by extraction five times in 80% (v/v) ethanol at 80°C, with intermittent centrifugation at 10 000 *g* for 5min. The powder was suspended in 500 µl of 100mM Na citrate buffer (pH 4.4) and incubated for 5min at 80 °C to gelatinize the starch. Heat-stable α-amylase (0.1 KNU-T units; Termamyl, Novozymes, Denmark) was added and the samples were incubated for 6h at 80°C. The tubes were cooled to 60°C and 0.3 AGU units of amyloglucosidase (Dextrozyme, Novozymes, Denmark) were added to samples. Samples were incubated at 60°C overnight, with constant mixing. After centrifugation, the supernatant was analysed for glucose using high-performance anion-exchange chromatography, with a DX 500 system (Dionex, Sunnyvale, CA, USA) equipped with an S-3500 auto sampler, GP40 pump, ED40 PAD (HPAEC-PAD), and a CarboPac PA-1 column as previously described (Blennow *et al.*, 2000b)

Starch measurements in leaves were performed, with modifications, as described in (Scofield *et al*., 2009). *Brachypodium* line 21-3 was used for sampling. Leaf samples were collected over a 14-h light period every 2h, immediately frozen in liquid nitrogen and stored at –80°C. The tissue was ground to fine powder using a tissue lyser. Samples of 20mg were used for analysis. Soluble sugars were removed by extraction four subsequent times in 80% (v/v) ethanol at 80°C for 1h followed with intermittent centrifugations at 13 000 *g* for 5min. The powder was suspended in 200 µl of 0.2M NaOH, incubated for 30min at 80°C to gelatinize the starch, and neutralized with 15 µl of 1M HCl. Na acetate buffer (200 µl; 20mM, pH 4.8) containing α-amylase (0.1 units, Sigma Chemicals) and amyloglucosidase (4 units, Sigma Chemicals) was added and the samples incubated at 50°C overnight with constant mixing. The supernatants were analysed as for the grain samples.

### Staining for starch in leaves

Leaves from *Brachypodium* (line 21-3) and barley were collected after a 10-h light period or a 14-h dark period. Pigments were removed by extraction in 80% (v/v) ethanol at 80°C and the destained leaves were transferred to petri dishes and stained with iodine solution.

### Amylose content

The apparent amylose content in starch granules was determined by iodine colorimetry (Wickramasinghe *et al.*, 2009). Each sample (2mg) was dissolved in 250 µl of 4M NaOH and incubated for 4h with gentle mixing. 750 µl of H_2_O was added to each sample and 10 µl aliquots applied in wells of a 96-well microtiter plate in technical triplicates. 200 µl of diluted iodine solution [stock of 2.6% I_2_ (w/v), 26% KI (w/v), diluted ×1000 in 1M HCl] was added, the absorbance measured at 550nm and 620nm and amylose concentration calculated by plotting the A620/A550 ratio against standards with known amylose contents (Carciofi *et al.*, 2011).

### Amylopectin chain-length distribution

Analysis of the distribution of the amylopectin side chains was performed from starches isolated from grain and leaf tissue of *Brachypodium* and barley. Commercially available grain starch from rice (*Oryza sativa* L. ssp. *indica*) was used as a control. Each sample (1mg) was incubated with 1M NaOH at 80°C for 5min and the pH was neutralized with 10 µl 1M HCl. Citrate buffer (100 µl; 0.1M) was added containing 1 µl of isoamylase (Sigma Chemicals), samples were incubated for 1h at 40°C, shortly centrifuged, and supernatants were analysed by high-performance anion exchange chromatography with pulsed amperometric detection (HPAEC-PAD) using a CarboPac PA-100 column as described previously (Blennow *et al.*, 2000b).

### Differential scanning calorimetry

Starch granule thermal characteristics in water were analysed using differential scanning calorimetry (DSC) as described previously (Carciofi *et al.*, 2011) using a Perkin Elmer Diamond DSC operated from 30°C to 100°C at a scanning rate of 10 °C min^–1^. The starches extracted from endosperm were analysed in slurries of 3mg native starch and 12 µl 10mM NaCl in technical triplicates. The endotherm transition was identified and the Perkin Elmer Pyris 7.0 software used to determine the parameters onset temperature (T_O_), peak temperature (T_P_), conclusion temperature (T_C_), and enthalpy transition energy (ΔH).

### X-ray powder diffraction

X-ray diffraction (XRD) analyses were performed as described previously (Tawil *et al.*, 2011).

###  Glucose-6-phosphate and glucose-3-phosphate content

The degree of starch phosphorylation was determined as the content of Glucose-6-phosphate (Glc-6-P) and glucose-3-phosphate (Glc-3-P) after complete starch hydrolysis as described previously (Blennow *et al.*, 1998) using an HPAEC-PAD system fitted with a CarboPac PA-1 column. Concentrations were determined using standard potato starches with known Glc-6-P and Glc-3-P content as determined by NMR (Blennow et al., 2000a, 2000b).

### Scanning electron microscopy

Grains were sectioned (400 µm) and dry starch granules previously purified from *Brachypodium* and barley grains were mounted on carbon tabs on aluminium stubs. Samples were sputter coated with gold/palladium for 120 seconds and observed with a secondary detector at an accelerating voltage of 10kV in Quanta 200 scanning electron microscopy (SEM; FEI Company, Eindhoven, Netherlands).

### Bright field and polarised light microscopy

Sections of 400 µm thickness of both *Brachypodium* and barley grains were produced using a microtome with vibrating blade Microm HM 650 V-Thermo Scientific. The slices were stained with iodine solution (as above) and observed with light microscopy using a Leica DMR HC fluorescence microscope combined with digital camera Leica DC 300F. Shape and size of extracted starch granules from seeds and leaves were investigated using bright field (BF) microscopy after suspending powder in iodine solution. Granule birefringence was investigated using a cross-polarizer.

### Confocal laser scanning microscopy

Native starch granules were stained with 8-amino-1,3,6-pyrenetrisulfonic acid (APTS) and afterwards confocal laser scanning microscopy (CLSM) was performed as described previously (Blennow *et al.*, 2003).

### Starch particle size analysis

For *Brachypodium*, 10 light micrographs of 600 iodine-stained starch granules were recorded and the granules were measured using ImageJ software (Schneider *et al.*, 2012). The size distribution for barley starch was measured by laser diffraction using a Microtrac S3000 analyzer (Microtrac, PA, USA). Starch granules (50mg) were suspended in ddH_2_O and sonicated for 10min before analysis to avoid aggregation. The data was analysed using Microtrac Flex Software. For both *Brachypodium* and barley, granule size distribution was calculated on the basis of number and volume frequency.

### (1–3)(1–4)-β-glucan content

(1–3)(1–4)-β-glucan analysis was performed according to Guillon *et al.* (2011) using a Megazyme mixed-linkage β-glucan kit (AOAC method 996.11; (1–3)(1–4)-β-glucan, AOAC method 995.16) with the modification that quantification of glucose was done using HPAEC-PAD chromatography (Blennow *et al.*, 2000b).

### Total nitrogen and protein

Total nitrogen and carbon contents of the grains were determined on finely ground grains using an ANCA-SL/GSL elemental analyser (SerCon, UK) attached to a stable isotope mass spectrometer. A nitrogen-to-protein conversion factor of 5.7 was used for protein content (Mosse, 1990).

## Results

### *Brachypodium* possesses full genetic capacity for starch biosynthesis

The comparative genetic capacity for starch biosynthesis and degradation in *Brachypodium* and barley was investigated *in silico* and the enzyme functional gene structure deduced. We identified 23 unique genes and annotated them in the groups starch synthases (SSs), starch-branching enzymes (SBEs), debranching enzymes (DBEs), α-glucan phosphorylase (PHOs), and glucan water dikinases (GWDs) according to their catalytic domains. Recent annotations (Li *et al.*, 2012; Trafford *et al.*, 2013) were confirmed with updates for BdAPL1, BdSSIIIa, BdSBEIII, ISAIII, and BdGWDs, while ADP-glucose pyrophosphorylase (BdAGPase) genes were identified previously (Comparot-Moss and Denyer, 2009). GWDs were included to provide a comprehensive comparative list of *Brachypodium* and barley genes for starch metabolism (Table 1). The number of identified paralogues for DBEs, GWDs, SBE,s and PHOs were identical for *Brachypodium* and barley. *Brachypodium* had additional SSII and AGP-L paralogues. These results demonstrate that the genetic capacity for starch metabolism is largely conserved through grass evolution and domestication.

**Table 1.** Comparative list of the *in silico* predicted starch biosynthesis and degradation proteins in *Brachypodium* and barley genomes^a^

Enzyme name and abbreviation			Purple false brome	Barley	Reference for barley
			*Brachypodium distachyon*	*Hordeum vulgare*	
ADP-glucose pyrophosphorylase AGPase (EC 2.7.7.27)	Small subunit	AGP-S	BdAPS1 cyt	AGP-S1 cyt	Radchuk *et al.* (2009); Huang *et al.* (2011) Comparot-Moss and Denyer (2009)
			Bradi3g22330	CAA88449	
			BdAPS2	AGP-S2	Radchuk *et al.* (2009); Huang *et al.* (2011); Comparot-Moss and Denyer (2009)
			Bradi4g27570	AAO16183	
				AGP-S3	Huang *et al.* (2011)
				CAX51352	
	Large subunit	AGP-L	BdAPL1	AGP-L1	Radchuk *et al.* (2009); Huang *et al.* (2011)
			Bradi1g09537	CAA47626	
			BdAPL3	AGP-L2	Radchuk *et al.* (2009); Huang *et al.* (2011)
			Bradi2g14970	AAC49729	
			BdAPL4		
			Bradi1g53500		
Starch synthase (EC 2.4.1.21)	Granule-bound starch synthase	GBSS	GBSSI	GBSSIa	Radchuk *et al.* (2009)
			Bradi1g50090	AAM74051	
			GBSSII	GBSSIb	Radchuk *et al.* (2009)
			Bradi2g41590	AAM74054	
			GBSSIb		
			Bradi4g00650		
	Soluble starch synthase	SSI	SSI	SSI	Radchuk *et al.* (2009)
			Bradi1g48610	AAF37876	
		SSII	SSIIa	SSIIa	Li *et al.* (2003)
			Bradi1g45130	AAN28309	
			SSIIb	SSIIc	Yan *et al.* (2009)
			Bradi3g59440	AK251488 (nucleotide)	
			SSIIc		
			Bradi3g27260		
		SSIII	SSIIIa	SSIIIa	Radchuk *et al.* (2009)
			Bradi3g15027	HB14E10 (EST)	
			SSIIIb	SSIIIb	Radchuk *et al.* (2009)
			Bradi5g22310	HB14B08 (EST)	
		SSIV	SSIV	SSIV	Radchuk *et al.* (2009)
			Bradi2g18810	HF05C15 (EST)	
Starch-branching enzyme (EC 2.4.1.18)		SBEI	SBEI	SBEI	Radchuk *et al.* (2009)
			Bradi1g29850	AAP72268	
		SBEII	SBEIIa	SBEIIa	Radchuk *et al.* (2009); Hazard *et al.* (2012)
			Bradi5g09170	AAC69753	
			SBEIIb	SBEIIb	Radchuk *et al.* (2009); Hazard *et al.* (2012)
			Bradi3g44760	AAC69754	
		SBEIII	SBEIII	NF	
			Bradi1g41970		
Starch-debranching enzyme	Isoamylase (EC 3.2.1.68)	ISA	ISA1	ISA1	Radchuk *et al.* (2009)
			Bradi3g40410	AAM46866	
			ISA2	ISA2	Radchuk *et al.* (2009)
			Bradi2g26170	BAD08581	
			ISA3	ISA3	Radchuk *et al.* (2009)
			Bradi4g32707	BAD89532	
	Limit dextrinase (EC 3.2.1.41)	PUL	PUL	PUL	Radchuk *et al.* (2009)
			Bradi5g00540	AAD34348	
α-Glucan phosphorylase (2.4.1.1)		PHO	PHO1	PHO1	Radchuk *et al.* (2009)
			Bradi1g08070	HB21H16 (EST)	
			PHO2	PHO2	Radchuk *et al.* (2009)
			Bradi2g55120	HO06A20 (EST)	
Glucan water dikinase (EC 2.7.9.4)		GWD	GWD1	GWD1	Radchuk *et al.* (2009)
			Bradi3g11270	HO15I19 (EST)	
			GWD2	GWD2	Radchuk *et al.* (2009)
			Bradi1g41907	HDP27A11 (EST)	
			GWD3/PWD	GWD3/PWD	Radchuk *et al.* (2009)
			Bradi1g50530	HVSMEl0024A09f (EST)	

^a^ Barley enzymes are given with GenBank accession numbers, and *Brachypodium* orthologues are given with gene identifiers.

In order to identify the main starch biosynthetic relationships in *Brachypodium* compared with major members of the Poaceae family and selected well-characterized dicotyledonous plants we constructed a phylogenetic tree with these members based on the starch synthase protein sequences (Supplementary Figure S1). Each clade turned out to constitute a cluster with specific enzymatic function, experimentally documented for many members. The putative *Brachypodium* BdGBSSIb sequence is an outlier. Its sequence codes for a catalytic domain, but the protein sequence is possibly truncated indicating that it might be a pseudogene.

**Fig. 1.**
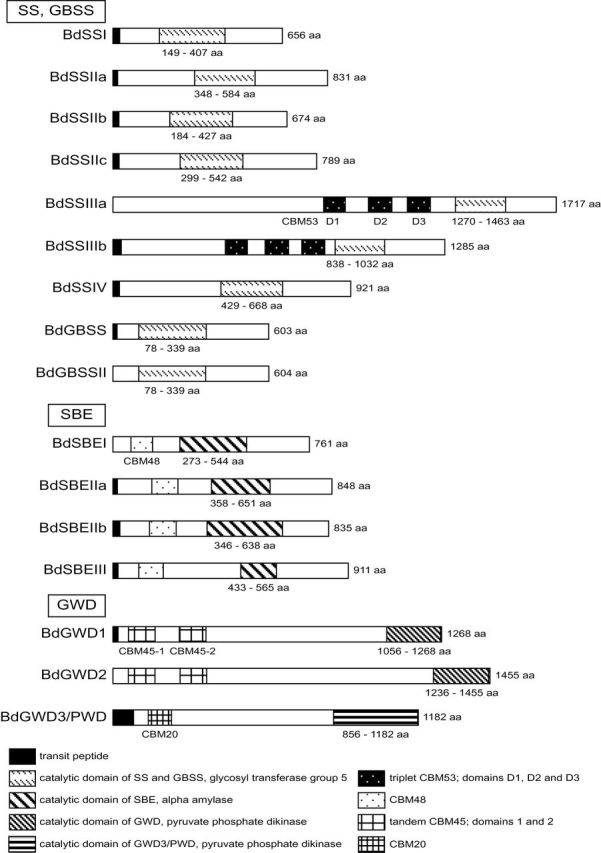
Domain structure of selected enzymes in starch biosynthesis and degradation, showing transit peptides, domains of CBMs, and catalytic domains. The size of the proteins is given in amino acids (aa). Genes of starch synthesis: SS, starch synthases; GBSS, ground-bound starch synthase; SBE, starch-branching enzymes; GWD, glucan water dikinase; PWD, phospho glucan water dikinase.

The functional domain structure of transferase orthologues are expected to be well conserved across the Poaceae family and we therefore identified the SS catalytic domain, non-catalytic carbohydrate-binding module (CBM) with affinity to starch and plastid-targeting signal peptides (Fig. 1). Four different CBMs were identified and positioned in the sequences belonging to the CBM family CBM20 (Christiansen *et al.*, 2009a, b), CBM45 (Mikkelsen *et al.*, 2006), CBM48 (Machovič and Janeček, 2008), and CBM53 (Christiansen *et al.*, 2009a). In SSIII three internal repeats of CBM53 were present in the middle part of the coding sequence. These domains have been suggested to have a regulatory role (Valdez *et al.*, 2008). CBM45 is present as a tandem repeat domain present in GWD1 and GWD2 (Glaring *et al.*, 2011) and these are indicated to determine the lengths of the chains to be phosphorylated (Mikkelsen *et al.*, 2006). CBM48, typical for SBEs, was identified in BdSBEs, and CBM20 was found in the BdPWD/GWD3 (Christiansen *et al.*, 2009b).

### *Brachypodium* and barley starch genes have different gene expression patterns

Comparative expression profiling of genes involved in starch biosynthesis in *Brachypodium* and barley was carried out based on available data (Radchuk *et al.*, 2009; Davidson *et al.*, 2012) (Supplementary Figure S2). Distinct gene expression was noted for most of the genes.

Development of *Brachypodium* grain takes less time than of that of barley. Cellularization starts at 8 DAP for *Brachypodium* and 6 DAP for barley; the post-cellularization period ends with maturity at 20 DAP and 38 DAP, respectively (Trafford *et al.*, 2013). Typically, gene expression in *Brachypodium* was high at 5 DAP, while still at the pre-cellularization stage, and declined towards endosperm maturation. In barley, expression was typically low and induced at the pre-cellularization stage, around 5 DAP, then increased considerably after cellularization started, rising at the later stages of endosperm maturation, and peaking at ~10–16 DAP and 26 DAP. The *ssIV* gene is a notable example showing distinct differences in expression dynamics at the pre-cellularization stage, at 5 DAP and 25 DAP, when *Brachypodium* grains are already mature. In *Brachypodium* the *ssIV* gene had declining expression, while the opposite was observed for barley. It is known that the *ssIV* gene in *Arabidopsis* controls the number of starch granules per chloroplast, through involvement in starch granule initiation (Roldán *et al.*, 2007). The striking decrease in *ssIV* expression in *Brachypodium* indicates that starch granule initiation is less important at later stages, which is also documented by the presence of only two types of granules in *Brachypodium*. Genes *ssIIIa* and *isa2* had similar profiles, except for *Brachypodium* in which expression did not decline further after 10 DAP, but had slightly increased at 25 DAP. Genes *agp-l1*, *gbssIa*, *sbeIIa*, *pho1*, *isa1*, *agp-s2*, *ssI*, *ssIIa*, *sbeIIb*, and *pho2* in *Brachypodium* showed maximum expression at 5 DAP after which the level of gene expression declined. In barley the same genes peaked around 10–16 DAP and then expression declined (*agp-l1*, *gbssIa*, *sbeIIa*, *pho1*, *ssI*, *ssII*, and *sbeIIb*) or remained at the same high level (*agp-s2*, *isa1*). The *sbeI* gene was the only one in *Brachypodium* having constant, slightly increasing expression, while in barley it increased extensively after 10 DAP to peak at 15 DAP, followed by a decrease to half maximum expression level.

### *Brachypodium* grains are high in BG and protein content

Total grain starch and BG contents were determined in *Brachypodium* and barley (Table 2). *Brachypodium* grains contained very high amounts of BG, around 29.3% in Bd21 and 33.2% in Bd21-3 (Table 2). Hence, there was little difference found in grain composition between the two *Brachypodium* lines. However, *Brachypodium* had distinctly higher BG and protein content at the expense of starch when compared to barley. The protein content, as calculated from total nitrogen, was 28.6% in Bd21, 28.5% in Bd21-3 lines, and 12.3% in barley; and total starch was 12.3% and 11.1% in lines Bd21 and Bd21-3, respectively, which was 4-fold lower than in barley (47.1%).

**Table 2.** Grain and starch granule composition in two lines of *Brachypodium* and barley

	Bd 21	Bd 21-3	Barley GP
**Grain composition**			
Protein content (%)	28.57	28.53	12.26
Total starch content (%)	12.30±0.87	11.10±2.27	47.10±0.83
Mixed beta glucan content (%)	29.30±0.77	33.20±1.71	4.10±0.84
**Starch granule composition**			
Starch amylose content (%)	34.0±1.8	34.5±1.0	29.9^c^
Starch phosphate content (nmol Glc-6-P mg^–1^)	0.40±0.05	0.35±0.03	0.81±0.37^b^
Starch phosphate content (nmol Glc-3-P mg^–1^)	0.15±0.02	0.12±0.01	0.32^b^

^a^ SD of at least triplicates is indicated. ^b^ Carciofi *et al.* (2011). ^c^ Carciofi *et al.* (2012).

### *Brachypodium* starch is branched as is typical for temperate grasses, but granules are distinct

**Fig. 2.**
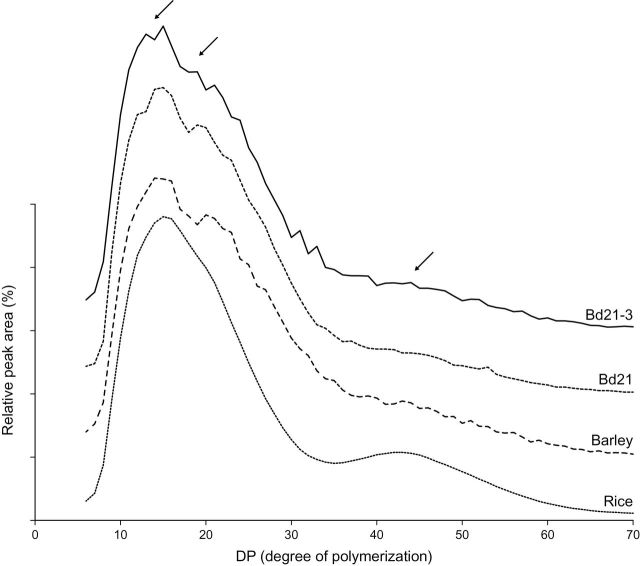
Chain-length distribution of amylopectin from *Brachypodium* lines 21 and 21-3, barley, and rice. Arrows indicate peaks at DP 14, DP 19-20, and DP 45.

In order to study the molecular composition of the starch of *Brachypodium*, amylose, starch-phosphate (Table 2), and amylopectin chain-length distribution (Fig. 2) were analysed in pure starch. Moreover, since molecular structure probably affects starch granule organization, the starch granule size distribution, morphology, topography, and crystallinity were investigated.

The amount of amylose in *Brachypodium* starch (34%) was higher compared to the level found in barley starch (29%). On the other hand, *Brachypodium* starch contained 2-fold less phosphate (0.35–0.40 nmole Glc-6-P mg^–1^ and 0.12–0.15 nmole Glc-3-P mg^–1^) as compared to barley (0.80 nmole Glc-6-P mg^–1^ and 0.32 nmole Glc-3-P mg^–1^).

The amylopectin chain-length distribution, as compared to rice and barley (Carciofi *et al.*, 2012) (Fig. 2) showed a polymodal distribution with distinct chain populations peaking at degree of polymerization (DP) 14 and DP 40–45. These distributions are fully consistent with those generally found in the plant kingdom (Blennow *et al.*, 2000b). However, interesting fingerprints specific for *Brachypodium* and barley were found in the DP 14–20 region where both *Brachypodium* and barley had a distinct peak at DP 19–20, which was lacking in the rice distribution.

**Fig. 3.**
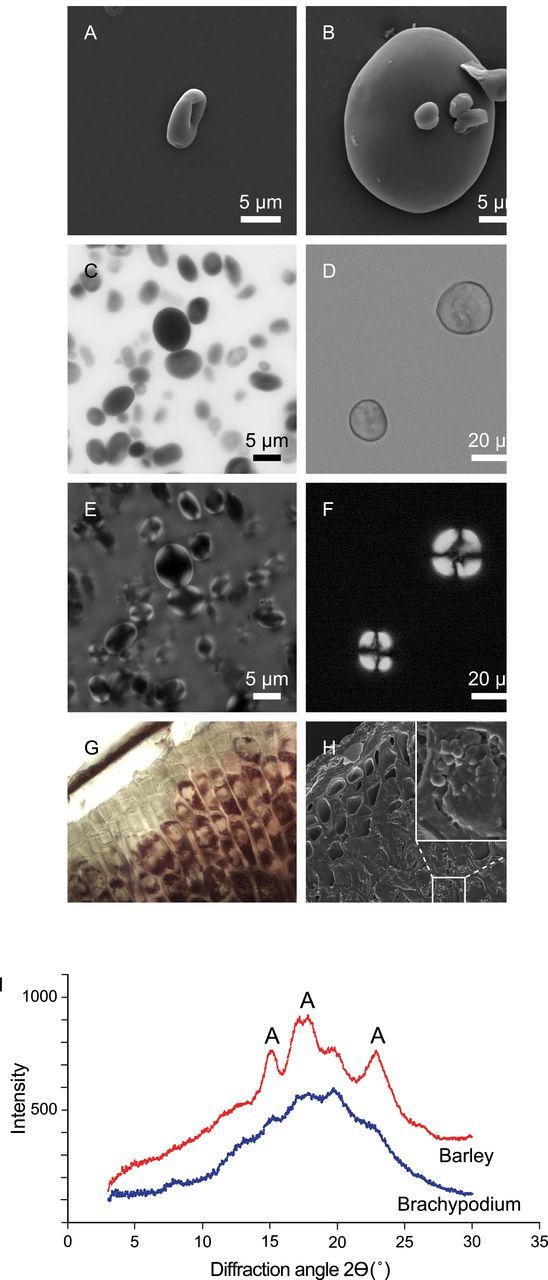
Analysis of starch granules in *Brachypodium* and barley: Scanning electron micrographs of (A) *Brachypodium* starch granule and (B) barley starch granules, indicating large A-type and small B-type granules. Bright field micrographs of (C) *Brachypodium* and (D) barley starch granules. Polarization light micrographs of (E) *Brachypodium* and (F) barley starch granules showing difference in birefringence. Starch granules in *Brachypodium* grains (G–H) are located only in the endosperm and are not present in the aleurone layer. XRD (I) of *Brachypodium* (bottom) and barley (top) starch granules; the diffraction peaks at 2Θ typical for the A-type crystalline polymorph are indicated. This figure is available in colour at *JXB* online.

The shape and size of *Brachypodium* starch granules as studied by light microscopy and SEM were examined and compared to barley granules (Fig. 3A, B). Light microscopy was used to study granule shape, while SEM gave more insight about the topography and surface of starch granules (Fig. 3C, D). The shapes of the *Brachypodium* granules were round to ellipsoid, but many were flattened, appearing as concave disks with depressions in the centre forming doughnut-shaped structures. Their surfaces were smooth, just like barley granules. Pores on the surfaces of *Brachypodium* granules could not be observed, suggesting that they do not exist or are too small to be seen. In the *Brachypodium* grain, we confirmed that starch was exclusively found in the endosperm cells (Fig. 3G, H) (Opanowicz *et al.*, 2011; Trafford *et al.*, 2013).

Anisotropic orientation of glucan chains in the starch granule, as deduced from polarization microscopy of *Brachypodium* grain starch granules (Fig. 3E, F), shows birefringence indicating that the molecular orientation was probably radial (Glaring *et al.*, 2006). The majority of starch granules of *Brachypodium* only showed birefringence in the peripheral 1–3 µm layer, indicating lower levels of orientation of polymer chains in the central granule region than barley starch granules which show clear birefringence.

**Fig. 4.**
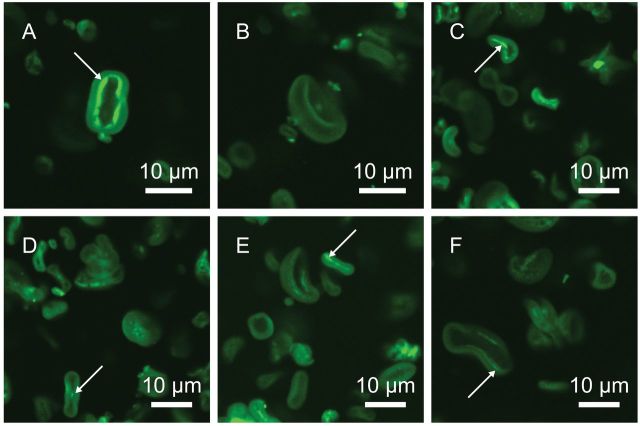
Confocal laser scanning micrographs of purified starch granules from *Brachypodium* line 21-3 stained with APTS. Arrows indicate amylose-rich areas. This figure is available in colour at *JXB* online.

The internal structures of the *Brachypodium* starch granules were investigated using CLSM (Fig. 4A–F) giving a more comprehensive insight into the internal microstructure of the grain storage starch granules. A discoid shape and depressions were clearly visible in the granules of all sizes, but pores, channels, or growth rings were not evident. The fluorescent probe APTS is linked to the reducing ends of each polysaccharide and hence amylose, being considerably smaller, generated the most fluorescence. White arrows on the micrographs indicate amylose-rich areas of *Brachypodium* granules. The inner structure of *Brachypodium* granules was similar to the structure of barley granules (Shaik *et al*., manuscript in preparation); even if the outer shape was different, barley granules were more oval and filled, while *Brachypodium* granules kept the doughnut-shape.

**Fig. 5.**
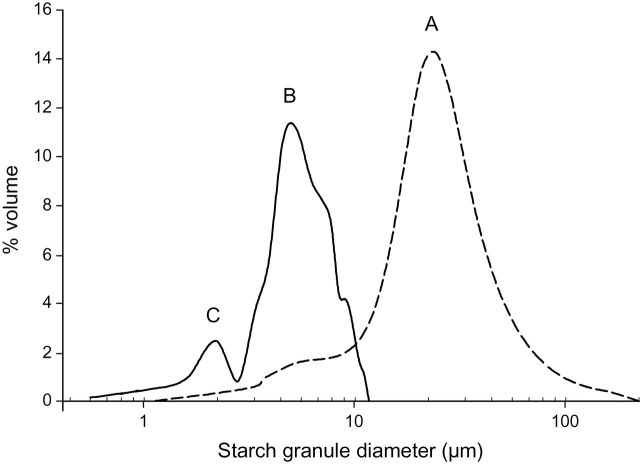
Starch granule size distribution of *Brachypodium* line 21-3 (solid line) and barley (dashed line). The A-, B-, and C-types of starch in barley are indicated. The B- and C-type populations of *Brachypodium* coincide with the barley B- and C-type granules.

As measured by image analysis, the estimated size of the *Brachypodium* endosperm starch granules ranged between 0.5 and 12 µm in *Brachypodium*, displaying a bimodal size distribution (Fig. 5). The two classes are small (2.5–10 µm) granules and very small (0.5–2.5 µm) granules, which according to Bechtel (1990) would correspond to B- and C-type granules. Seemingly, *Brachypodium* did not possess starch granules comparative to large A-type granules in barley. Starch granules in barley showed a typical bimodal distribution consisting of larger ≥15 µm A-type granules and smaller 2–5 µm B-type granules (Fig. 5) (Jane *et al.*, 1994).

The crystalline polymorph and degree of crystallinity were investigated for isolated *Brachypodium* and barley starch granules by powder X-ray crystallography (Fig. 3I). *Brachypodium* starch showed an A-type crystalline polymorph (diffraction peaks at 2Θ at 15.1°, 17.8°, and 23°), which is typical for cereal storage starch. However, the wide-angle X-ray scattering (WAXS) diagram of *Brachypodium* contains traces of V-type crystalline polymorph (diffraction peaks at 2Θ at 7°, 13°, and 20°). The degree of crystallinity was low and estimated to be 10%, which is two times lower than that observed for barley starch (20%) (Carciofi *et al.*, 2012).

Crystalline packing as analysed by dissolution in aqueous medium analysed by DSC revealed that the enthalpy of melting of *Brachypodium* starch was 2.72 and 3.10 J g^–1^ for Bd21 and Bd21-3, respectively and 6.32 J g^–1^ for barley (Table 3), supporting the more disordered structure of the *Brachypodium* starch granules as compared to barley starch granules.

**Table 3.** Thermal properties for two lines of *Brachypodium* and barley: the melting enthalpy (ΔH), onset (T_O_), peak (T_P_), and conclusion temperature (T_C_)^a^

	Bd 21	Bd 21-3	Barley GP^b^
ΔH (J g^–1^)	2.7±0.2	3.1±0.1	6.3±0.2
T_O_ (°C)	54.5±0.8	54.0±0.2	61.4±0.4
T_P_ (°C)	59.9±0.1	59.8±0.2	66.0±0.1
T_C_ (°C)	67.6±0.1	65.4±0.1	70.1±0.0

^a^ SD of at least triplicates is indicated. ^b^ Carciofi *et al.* (2011).

### Transient starch in *Brachypodium* is higher than in barley

**Fig. 6.**
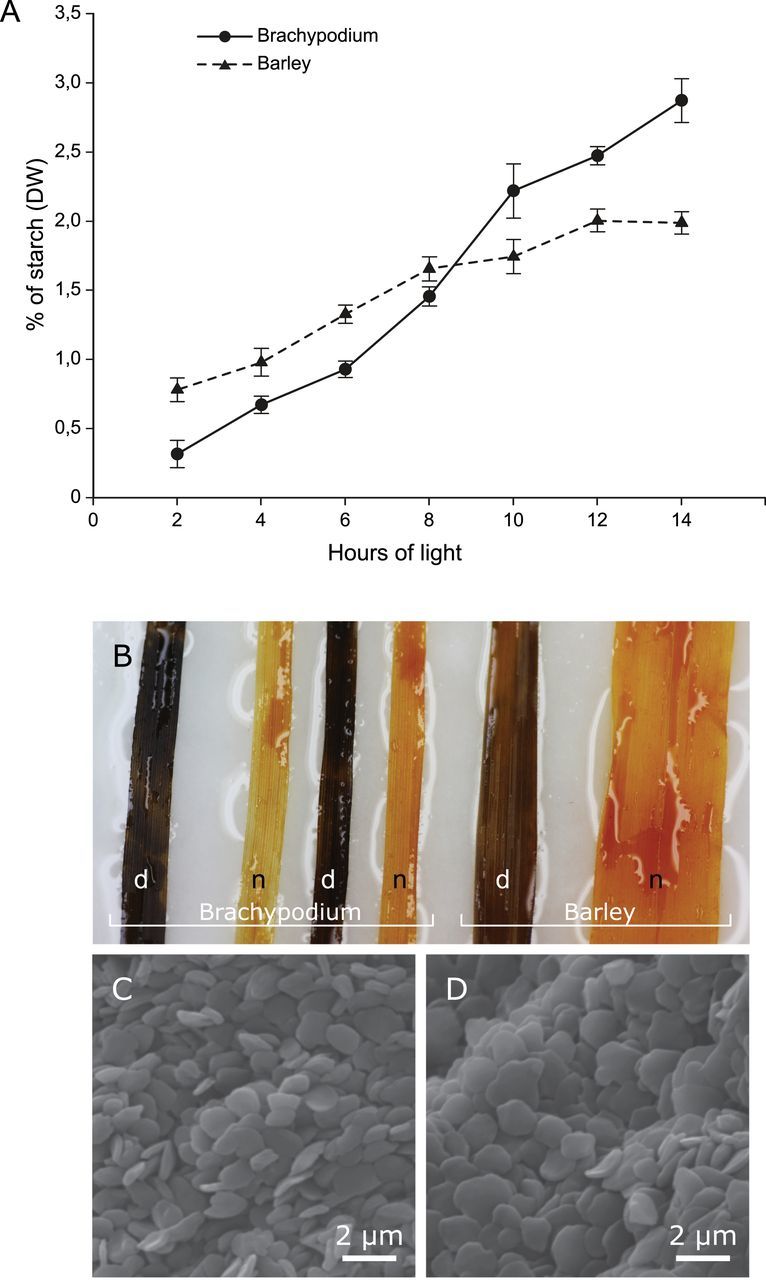
(A) Changes of starch content in leaves (DW) in *Brachypodium* line 21-3 (solid line) and barley (dashed line) during the light regime. (B) Leaves of *Brachypodium* and barley stained with iodine after a 24-h night (n) and a 10-h day (d). Shape and size of starch granules extracted from leaves of (C) *Brachypodium* and (D) barley as visualized with SEM. SD of at least triplicates is indicated. This figure is available in colour at *JXB* online.

The starch content was measured in leaves of *Brachypodium* and barley over a 12h light period, from 2h to 14h of light exposure, every 2h. The rate of starch biosynthesis progressed virtually linearly after 14h of light, reaching 2.9%. Interestingly, the amount of starch in barley also progressed linearly, but more steeply. After an 8-h light period, deposition of starch ceased and resulted in a lower amount (2%) than in *Brachypodium* after a 14-h light regime (Fig. 6A). Iodine staining of leaves showed a slightly darker colour in *Brachypodium* as compared to barley, confirming a higher concentration of starch in *Brachypodium* (Fig. 6B), which is in accordance with the measured amount of starch after 10h of light. Comparative amylopectin chain-length distribution analysis of leaf starch from *Brachypodium* and barley showed no significant difference at the end of the 14-h light period (data not shown). The *Brachypodium* leaf starch granule shape, size, and topography were very similar to that of barley leaf starch, having small, flattened, and round-shaped morphology as deduced from SEM (Fig. 6C, D).

## Discussion

Our study comprises a thorough characterization of biosynthesis and deposited structures of starch in the grass *Brachypodium*. *Brachypodium* possesses all the genes required for starch synthesis. Important starch-binding CBM motifs are conserved. Hence, any differences found in starch content and structure are likely to be found at transcriptional or post-translational levels. Gene-expression dynamics cannot be directly translated into enzyme activity in the cell. However, such data provide the general long-term capacity for specific activities to be interpreted in terms of starch molecular structure. For *Brachypodium*, gene expression was generally low throughout the endosperm including the expression of GWD starch phosphorylase. Moreover, distinct patterns in gene expression of *Brachypodium* and barley were found and can possibly explain the lower starch biosynthesis. As an example, the low expression levels found for *ssI* and *sbeI* substantiates this view (Trafford *et al.*, 2013).

Some peculiarities found for *Brachypodium* including small size, more flat shapes, absence of large A-type granules, low crystallinity, higher molecular disorder, and isotropy compared to barley starch granules demonstrate profound differences in their biosynthesis. Interestingly *Brachypodium* starch granules show a high level of similarity to immature cereal granules (Borén *et al.*, 2008), suggesting that starch biosynthesis in wild grasses as compared to domesticated cereals is slower or even arrested before granules adopt true crystalline lamellae and the layers typical for cereal starch granules. The very small (under 2.5 µm) and small (2.5 to 10 µm) granule populations cannot directly be translated to the A-, B-, and C-type classification as typically done according to the size, shape, and timing of their initiation in the endosperm (e.g. Bechtel *et al.*, 1990; Stamova *et al.*, 2009). The morphology of *Brachypodium* granules as identified in this study is unique, and hence difficult to directly compare to spheroid- and lens-shaped granules typically found in starches in comparable species. In our study, only size was used to discriminate starch granule type and it remains to be shown whether the B- and C-type classification holds in the sense of development of the grain and biosynthetic mechanism. In wheat (Yin *et al.*, 2012) the larger A-type granules first appear at 3 DAP, while smaller B-type granules occur around 15 DAP and both grow in size until the grain matures. There have been indications of B-granules being synthesized in connection to, and dependent on, the presence of A-type granules (e.g. Glaring *et al.*, 2006). In *Brachypodium*, the C-type granules might originate by budding from the B-type granules, but this mechanism remains to be confirmed. Hence, prolonged granule maturation and the formation of second generations of granules in the shape of small B- or C-type granules do not seem to be domestication effects. B-type starch granules are present in the genus *Hordeum* (Baum and Bailey, 1987) as well as in most species of wild wheat (genus *Aegilops*), where a genetic marker for B-type granules was identified (Stoddard and Sarker, 2000; Howard *et al.*, 2011) indicating that the biosynthesis of B-type granules is not necessarily linked to domestication events.

In summary, the differences in starch granule size and morphology in *Brachypodium* as compared to barley may occur for many reasons. Main differences were found in developmental maturity of the starch granules, the slow starch biosynthesis linked to low expression levels, and the different expression profiles of genes. Different expression profiles can potentially result in dissimilar balance of enzyme activities. A completely different starch biosynthetic mechanism is unlikely because of the conserved features of the enzymes expressed.

The substantially different grain compositions of the *Brachypodium* grain as compared to barley grain – mainly lower levels of starch and higher levels of protein and BG in *Brachypodium* – support recent data (Larre *et al.*, 2010, Guillon *et al.*, 2011, Trafford *et al.*, 2013) even though the actual levels differ indicating that *Brachypodium* grain composition, as compared to the more robust barley, is subject to genotypic and/or conditional variation. The data also support the general notion (Blennow *et al.*, 2013; Fig. 2) that domestication has driven an increase in endospermic starch biosynthesis at the expense of protein, cell wall BG, and possibly other constituents resulting in high content of dietary and easily accessible carbohydrate. The cell walls in *Brachypodium* endosperm are tremendously thick (Fig. 4G, H) in agreement with its high BG content, and are far too stiff for food and feed purposes. Interestingly, the high levels of BG provide a grain high in dietary fibre, an important asset for modern food. As indicated (Trafford *et al.*, 2013) the increased BG:starch ratio can be a direct consequence of reduced activity of *Brachypodium* enzymes in starch biosynthesis as compared to domesticated barley.

In leaves of *Brachypodium*, starch granules were of the same size as for barley. Interestingly, the rate of starch synthesis in *Brachypodium* and barley was different, resulting in higher leaf starch content in *Brachypodium*. Fructans are the major storage carbohydrate in the vegetative tissues of grasses, including barley (Hendry, 1993). We cannot exclude a similar situation in *Brachypodium*, even though our results indicate that leaf starch in *Brachypodium* is of greater importance as an energy reserve than in barley. This indicates that leaf starch in *Brachypodium* can be of greater importance as an energy reserve to be utilized in varying growth conditions and climates in this wild grass than in barley. In conclusion, more thorough cross-species analysis of wild-grass grains is required to assess the potential of wild grasses like *Brachypodium* as genetic resources to introduce novel high-value traits into modern cereals.

## Supplementary material

Supplementary data can be found at *JXB* online.

Supplementary Figure S1. Phylogenetic tree based on predicted protein sequences of different starch synthases.

Supplementary Figure S2. Comparison of expression profiles of selected starch biosynthesis genes in *Brachypodium* and barley in developing endosperm.

## Funding

This work was supported by a University grant (VT), and The Danish Ministry of Science, Innovation and Higher Education (SLJ).

## Supplementary Material

Supplementary Data
