# Supplementary material for: The deposition and characterization of starch in Brachypodium distachyon
Source: J Exp Bot. 2014 Jul 23;65(18):5179–92. doi: 10.1093/jxb/eru276 (PMC4157704; doi:10.1093/jxb/eru276)
Supplement: Supplementary Data [file supp_65_18_5179__index.html]

The deposition and characterization of starch in Brachypodium distachyon — The deposition and characterization of starch in Brachypodium distachyon — Supplementary Data 

# The deposition and characterization of starch in *Brachypodium distachyon*

## Supplementary Data

Data files

**Files in this Data Supplement:**

- Supplementary Data - Supplementary Data
