# Supplementary material for: The deposition and characterization of starch in Brachypodium distachyon
Source: J Exp Bot. 2014 Jul 23;65(18):5179–92. doi: 10.1093/jxb/eru276 (PMC4157704; doi:10.1093/jxb/eru276)
Supplement: Supplementary Data [file supp_eru276_jexbot124362_file001.pdf]

Supplement 1. Phylogeny tree based on starch synthases predicted protein sequences from Arabidopsis thaliana (At), Solanum tuberosum (St), Hordeum vulgare (Hv), Oryza sativa (Os), Triticum aestivum (Ta), Zea mays (Zm), Brachypodium distachyon (Bradi), Chlamydomonas reinhardtii (Cr). Protein sequences used: At SSI, AAF24126; At SSII, AF26156; At SSIII, AAD30251; At SSIV, CAA16796; At GBSSI, NP_174566; St GBSSI, CAA41359; St SSII, CAA61241; St SSI, CAA71442; St SSIII, CAA64173; Hv SSI, AAF37876; Hv SSII, AAN28309; Hv GBSSI, AAM74051; Hv GBSSIb, AAM74054; Os GBSSII, BAC21549; Os SSI, AAP56350; Os GBSSI, AAF72561; Os SSII, AAL16661; Os SSIIb, AAK81729; Os SSIIIa, AAM49811; Os SSIIIb, AAL40942; Os SSIVa, AAQ82622; Os SSIVb, AAQ82623; Os SSIIC, BAF26592; Ta SSI, CAB99209; Ta SSIIa, CAB86618; Ta SSIIIa, AAF87999; Ta SSIIIb, ABY56823; Ta SSIV, AAK97773; Ta GBSSII, AAF14233; Ta GBSSI, BAA77351; Ta SSIIb, ABY56824; Ta SSIIc, ABY27639; Zm SSI, AAB99957; Zm SSII, AAD13341; Zm SSIIb-1, AAD13342; Zm SSIIb-2, ABP35814; Zm SSIIIb-1, ABP35815; Zm SSIIIb-2, ABP35816; Zm SSIIIa, AAC14014; Zm GBSSI, CAA27574; Zm BSSIIa, ABO71782; Zm SSIIc, ABX76967; Zm SSIV, ACC63897; Cr SSII, AAC17970; Cr SSIII, AAY42381; Cr SSIV, AAC17971. Sequences were collected from the National Centre for Biotechnology
168 Information database (NCBI).

Supplemental 1: Phylogeny tree

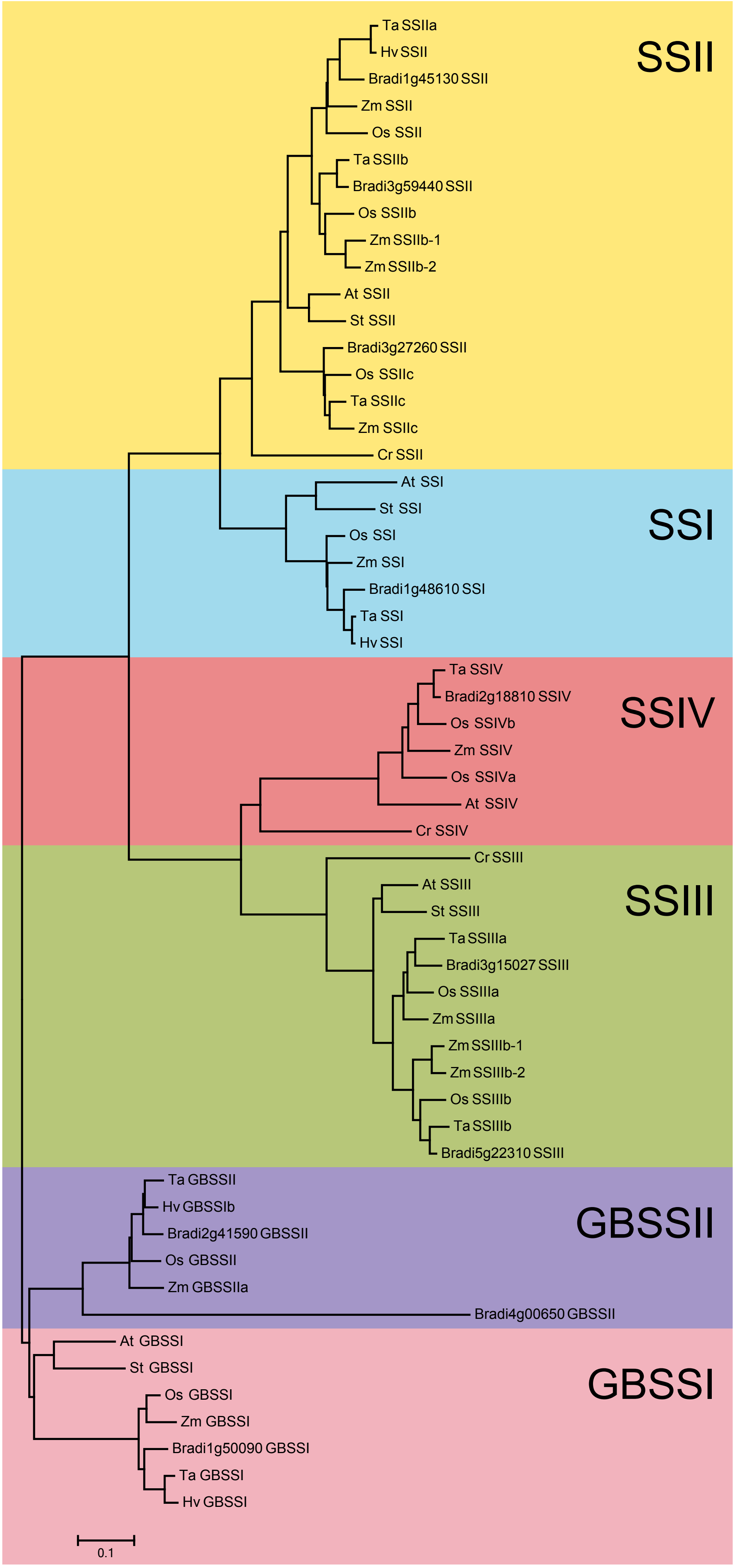

Supplement 2: Comparison of expression profiles of selected genes of starch biosynthesis, in Brachypodium (solid line) and barley (dashed line) in developing endosperm. Expression in Brachypodium was measured on 5, 10 and 25 DAP. In barley the same measurements were done starting with day 0, every two days, until 26 DAP. Transcriptome data for starch biosynthetic genes for Brachypodium and barley were extracted from previously published data sets obtained from Davidson (2012) and Radchuk (2010).

Supplemental 2: Expression profiles

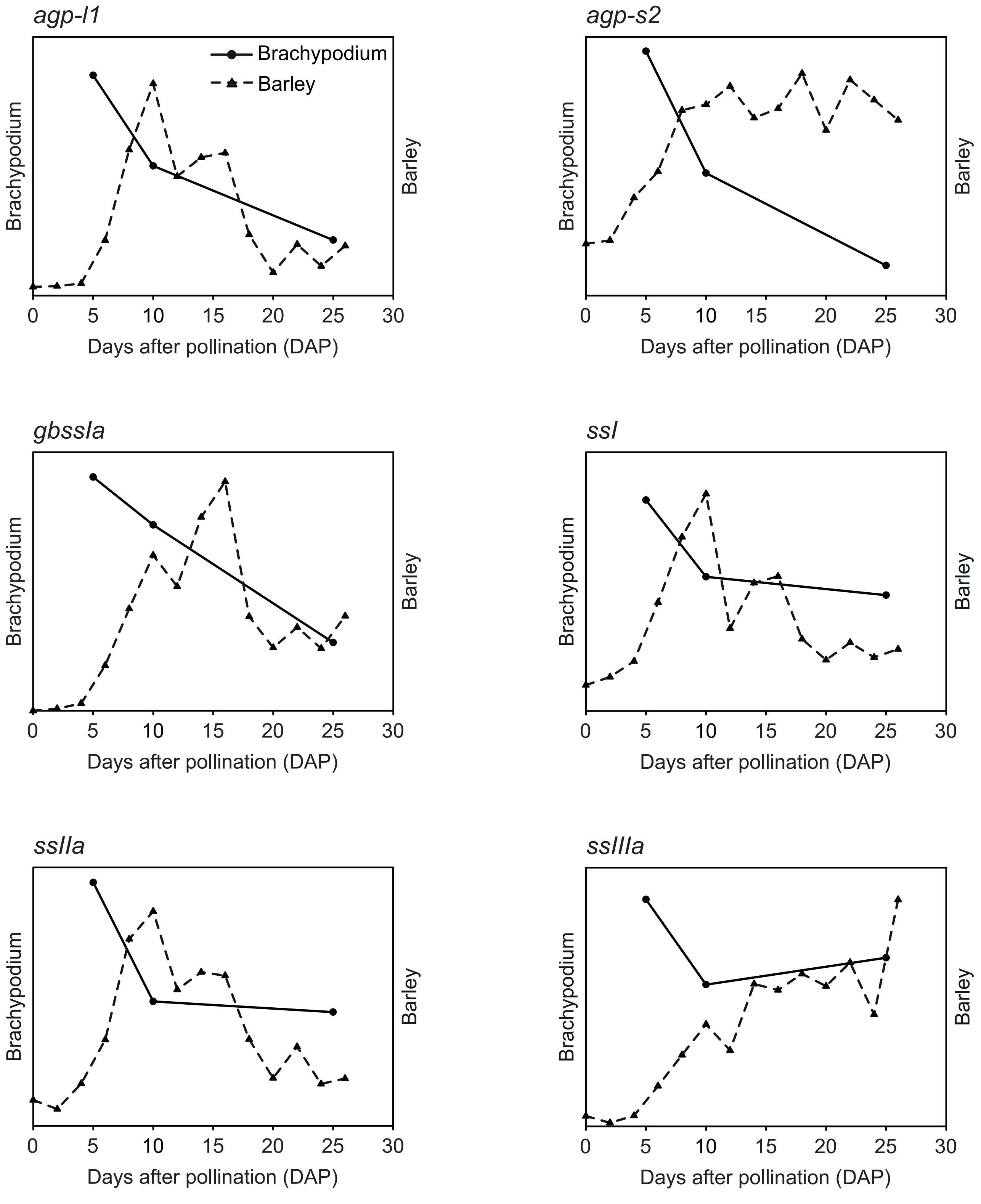

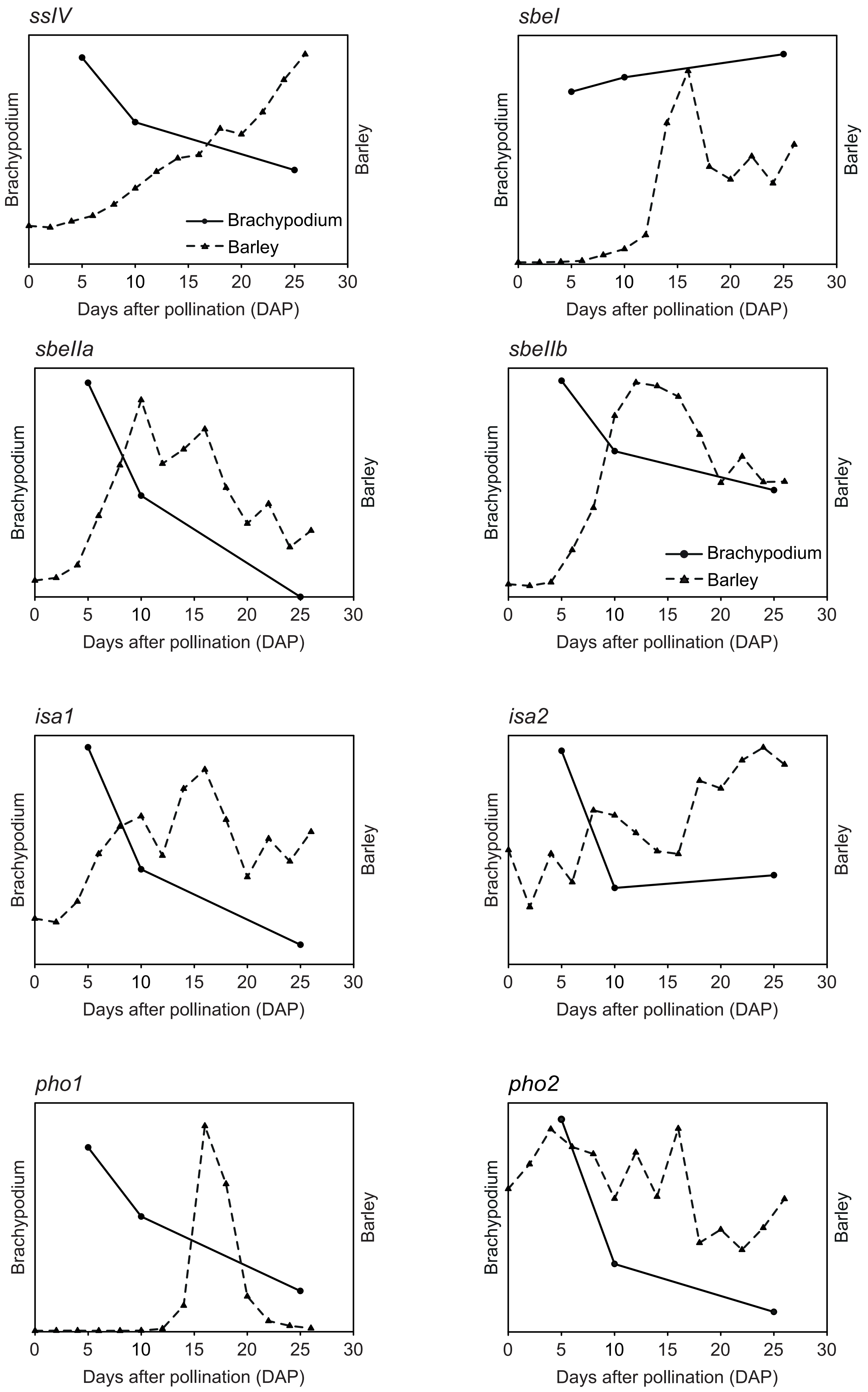
ssIV
sbeI
sbeIIa
sbeIIb
isa1
isa2
pho1
pho2
Brachypodium
Barley
Days after pollination (DAP)
0 5 10 15 20 25 30
